# Neuroprotective Effects of Oligodendrocyte Progenitor Cell Transplantation in Premature Rat Brain following Hypoxic-Ischemic Injury

**DOI:** 10.1371/journal.pone.0115997

**Published:** 2015-03-19

**Authors:** Long-Xia Chen, Si-Min Ma, Peng Zhang, Zi-Chuan Fan, Man Xiong, Guo-Qiang Cheng, Yi Yang, Zi-Long Qiu, Wen-Hao Zhou, Jin Li

**Affiliations:** 1 Department of Neonatology, Children’s Hospital of Fudan University, Shanghai, 201102, China; 2 Key Laboratory of Birth Defect, Children’s Hospital of Fudan University, Shanghai, China; 3 Key Laboratory of Neonatal Diseases, Ministry of Health, Children’s Hospital of Fudan University, Shanghai, China; 4 Institute of Neuroscience, Shanghai Institutes for Biological Sciences, Chinese Academy of Sciences, Shanghai, 200031, China; 5 Institutes of Biomedical Sciences, Fudan University, Shanghai, China; Massachusetts General Hospital/Harvard Medical School, UNITED STATES

## Abstract

Periventricular leukomalacia (PVL) is a common ischemic brain injury in premature infants for which there is no effective treatment. The objective of this study was to determine whether transplanted mouse oligodendrocyte progenitor cells (OPCs) have neuroprotective effects in a rat model of PVL. Hypoxia-ischemia (HI) was induced in 3-day-old rat pups by left carotid artery ligation, followed by exposure to 6% oxygen for 2.5 h. Animals were assigned to OPC transplantation or sham control groups and injected with OPCs or PBS, respectively, and sacrificed up to 6 weeks later for immunohistochemical analysis to investigate the survival and differentiation of transplanted OPCs. Apoptosis was evaluated by double immunolabeling of brain sections for caspase-3 and neuronal nuclei (NeuN), while proliferation was assessed using a combination of anti-Nestin and -bromodeoxyuridine antibodies. The expression of brain-derived neurotrophic factor (BDNF) and Bcl-2 was examined 7 days after OPC transplantation. The Morris water maze was used to test spatial learning and memory. The results showed that transplanted OPCs survived and formed a myelin sheath, and stimulated BDNF and Bcl-2 expression and the proliferation of neural stem cells (NSC), while inhibiting HI-induced neuronal apoptosis relative to control animals. Moreover, deficits in spatial learning and memory resulting from HI were improved by OPC transplantation. These results demonstrate an important neuroprotective role for OPCs that can potentially be exploited in cell-based therapeutic approaches to minimize HI-induced brain injury.

## Introduction

Brain injury in premature infants is a clinical syndrome associated with significant adverse outcomes [1]. Premature encephalopathy has multiple causes, including hypoxic-ischemia (HI), infection, physical trauma, and so on [2]; however, periventricular leucomalacia (PVL) is the predominant form of brain injury in premature infants, with no specific treatments currently available [3,4]. Survivors typically have neurodevelopmental sequelae, including cerebral palsy and cognitive or behavioral deficits, with a higher incidence observed in developing countries [3,5]. PVL primarily affects white matter, but accompanying damage to gray matter is also possible [6,7], resulting in demyelination and neuronal apoptosis; persistent demyelination is associated with axonal loss [8], which can produce permanent motor and sensory deficits.

Cell-based therapeutic approaches are an attractive option for treating PVL, with grafted cells not only replacing those that are lost, but providing neurotrophic benefits to surrounding tissue [9,10]. To date, transplanted NSCs, human umbilical cord blood mononuclear cells, and mesenchymal stem cells have been shown to survive and differentiate and improve functional recovery after HI injury [11,12,13,14,15]. Embryonic stem cells differentiated oligodendrocytes may become the preferred cell type for transplantations to treat white matter injury [16]. OPCs can differentiate into mature oligodendrocytes in vivo, which cover the axons forming myelin sheath. Multiple transplantation protocols have been studied in animal models for a variety of adult or term baby brain injury-related neurological disorders. However, few studies have been conducted on immature animal models and little work has been done on OPCs transplantation, except in a lipopolysaccharide-induced model of premature brain injury [7].

Although numerous studies have demonstrated the effectiveness of cell replacement therapy [17,18,19], few have investigated endogenous neuroprotection following transplantation. In order to gain insight into this process, OPCs were differentiated from a mouse (m)ESC line expressing GFP from the locus of Olig2, a transcription factor critical for OPC development [20]. Olig2^+^ GFP^+^ OPCs were transplanted into ventricular of premature rat brain subjected to HI. The current study investigated whether treatment of premature encephalopathy with OPCs can have a neuroprotective effect and improve cognitive function.

## Materials and Methods

### 2.1 Animal models

This study was conducted in accordance with the National Institutes of Health Guide for the Care and Use of Laboratory Animals. All protocols were approved by Clinical Pharmacology Ethics Committee of Fudan University, Pediatrics Ethics Committee in Shanghai. HI was induced in rats (7–9 g) at postnatal day 3 (P3), as previously described [1,21]. Briefly, pups of either sex were anesthetized with isoflurane, and the left common carotid artery was isolated and ligated with a 5–0 surgical silk suture. The procedure was completed within 5 min, and the pups were allowed to recover for 1 h in a temperature-controlled incubator. Animals were then placed in a container perfused with a humidified gas mixture (6% oxygen in nitrogen) at 36.5–37°C for 2.5 h. The pups were randomly assigned to one of two groups, HI+PBS or HI+OPC. Sham animals that underwent surgery but without artery ligation were randomly assigned to one of two control groups, S+OPC or S+PBS. A subset of animals from each group was tested in the Morris water maze 36–41 days after transplantation.

### 2.2 OPC preparation

Olig2-GFP mESCs were a gift from Dr. Su-Chun Zhang (University of Wisconsin, USA) and were differentiated according to a modified version of a previously published protocol [20]. Briefly, mESCs were trypsinized and transferred to low attachment flasks (Greiner Bio One, Germany) in a neural differentiation medium containing DMEM/F12, N2 supplement, leukemia inhibitory factor, nonessential amino acids, L-glutamine and 10% knockout serum replacement (all from Invitrogen, Carlsbad, CA, USA). The mESCs formed aggregates after 2 days, and retinoic acid (0.5 mM; Sigma, St. Louis, USA) and purmorphamine (0.5mM; Calbiochem, San Diego, USA) were added at days 2–6 to induce differentiation of neural progenitors. Cells were cultured as suspended aggregates in neural differentiation medium containing basic fibroblast growth factor (20 ng/ml; R&D Systems, USA) and heparin (2 mg/ml; Sigma) for 6 more days. On day 12, differentiated aggregates were dissociated with trypsin-EDTA (0.05%; Life Technologies, USA) and plated in Matrigel-coated flasks in modified Bottenstein-Sato medium containing insulin (10 μg/ml), bovine serum albumin (100 μg/ml), human transferrin (100 μg/ml), progesterone (60 μg/ml), sodium selenite (40 μg/ml), N-acetyl-cysteine (60 μg/ml), putrescine (16 μg/ml), biotin (10 ng/ml), and cAMP (5 μM) (all from Sigma). Triiodothyronine (40 ng/ml; Sigma), platelet-derived growth factor AA (10 ng/ml), and neurotrophin-3 (5 ng/ml) (all from R&D Systems) were added to stimulate OPC proliferation. OPC cells used in this study were provided by the Institutes of Biomedical Sciences of Fudan University (Shanghai, China) [22]. The purity and viability of transplanted cells were tested as previously described [22].

### 2.3 Cell transplantation by lateral ventricle injection

The pups were returned to their mother and allowed to recover for 2 h after HI. OPC suspensions were then injected into the left lateral ventricles of the pups in the HI+OPC and S+OPC groups, while an equivalent volume of PBS was injected into pups in the HI+PBS and S+PBS groups. Pups were placed in a stereotaxis under isoflurane anesthesia. An incision was made to the skin, and animals were injected through the skull with 2 μL OPC suspension (10^5^ cells/ μL) or 2 μL PBS through a needle over a period of more than 5 min, at the following coordinates from bregma: anteroposterior-1.0 mm; mediolateral-1.0 mm; dorsoventral-2.0~2.5 mm [7,23]. The syringe was left in place for an additional 5 min, then gradually withdrawn. The scalp was sutured and disinfected with 75% ethanol, and pups were returned and nursed by their mothers until the time of sacrifice. To label proliferating cells, 5-bromodeoxyuridine (BrdU), freshly prepared in PBS, was injected intraperitoneally (50 mg/kg) once daily after the transplantation procedure until the time of sacrifice.

### 2.4 Tissue preparation, immunohistochemistry, and imaging

Animals were deeply anesthetized at 2, 3, 7, 14 days and 6 weeks after transplantation, and perfused with 4% paraformaldehyde in 0.1 M PBS. Brains were removed and post-fixed in the same solution at 4°C for 24 h, then dehydrated in a graded series of sucrose (20% and 30%) until permeated. Coronal sections were cut on a freezing microtome (Jung Histocut, Model 820-II; Leica, Nussloch, Germany) at a thickness of 30 μm from bregma 1.60 to -4.80 mm, and stored at -20°C in cryoprotectant solution. Sections were incubated with mouse primary antibodies against brain-derived neurotrophic factor (BDNF, 1:200; ab108383; Abcam, USA) or Bcl-2 (1:200; Millipore, AB7973, USA) overnight at 4°C, followed by appropriate biotinylated secondary antibodies (1:200) and avidin-biotin-peroxidase (Vectastain Elite ABC kit; Vector Laboratories, USA) for 45 min at 37°C. Immunoreactivity was visualized with diaminobenzidine (DAB).

To detect OPC migration, survival, and differentiation, single brain sections were divided into quarters, and one quarter was selected for each type of staining. Rabbit anti-GFP antibody labeling (1:1000; AB3080, Millipore) was used to visualize transplanted cells. Apoptosis of OPCs was evaluated 6 weeks after transplantation. Sections were fixed in 4% paraformaldehyde for 30 min, then permeabilized for 20 min on ice before incubation with the terminal deoxynucleotidyl transferase dUTP nick end labeling (TUNEL) reaction mixture (Roche, 12156792910, USA) for 60 min at 37°C. Sections were blocked in goat serum at 37°C for 30 min, then incubated with anti-GFP antibody (1:1000; AB3080, Millipore) at 37°C for 2 h then at 4°C overnight. After washes with PBS, sections were incubated with fluorescein isothiocyanate (FITC)-conjugated anti-rabbit IgG (1:500; Invitrogen) at 37°C for 45 min.

For double immunolabeling for GFP/glial fibrillary acidic protein (GFAP), GFP/NeuN, GFP/myelin basic protein (MBP), BrdU/Nestin, and caspase-3/NeuN, sections were blocked in goat serum at 37°C for 30 min, then incubated with antibodies against BrdU (rat, 1:400; Santa Cruz Biotechnology, Inc., USA), GFP, or caspase-3 (rabbit, 1:20; BioVision, 3501–100, USA) at 37°C for 2 h, then at 4°C overnight. Sections were rinsed with PBS, and incubated with Cy3-conjugated anti-mouse or FITC-conjugated anti-rabbit IgG (both 1:500; Invitrogen) at 37°C for 45 min, followed by anti-Nestin (mouse, 1:100; MAB353, Millipore), anti-GFAP (mouse, 1:200; AB5804, Millipore), anti-MBP (mouse, 1:200;AB62631, Abcam), or anti-NeuN (mouse, 1:200; MAB377, Millipore) antibodies at 4°C overnight. After additional PBS washes, sections were mounted on glass slides with fluorescence mounting medium (Vector Laboratories, USA). Fluorescence was detected at excitation and emission wavelengths of 570/576 nm (Rhodamine) and 490/525 nm (FITC) using a Zeiss 510 confocal microscope (Carl Zeiss Microscopy, Thornwood, USA).

### 2.5 Morris water maze test

The water maze (Med Associates Inc., Georgia, USA) with dimensions of 1.6 m (diameter) × 50 cm (height), with the bottom 45 cm above floor level, was filled with water (22 ± 1°C). The black platform (12 cm diameter × 30 cm height) was submerged 1–1.5 cm below the water surface. The test was performed as previously described [24] 36–40 days after transplantation (n = 20 per group). For the navigation trial, rats underwent four trials daily with different starting points over four consecutive days. Rats escaping the platform were restricted within 120 s, and allowed to rest on the platform for 15 s. The space probe trial, with the platform removed, took place on the fifth day over 60 s. The escape latency, number of platform crossings, percentage of time spent in the platform quadrant, and percent distance traveled in the platform quadrant were recorded. Data were analyzed using a tracking program (DigBehv-MWM; Shanghai Jiliang Software Technology Co. Ltd., Shanghai, China).

### 2.6 Statistical analysis

Three consecutive immunostained tissue sections were selected for quantification. Double-labeled cells were analyzed by a computerized Stereo Investigator 6.5 (MicroBrightField Inc.,Williston, USA) using the optical-fractionator probe at 20× objective (n = 8–10 animals per group) [25]. DAB-positive cells were counted manually with Image Pro Plus 5.1 software (Media Cybernetics, Rockville, MD, USA). Data were initially analyzed with observers blinded to the experimental conditions. Data are expressed as mean ± SD. Differences between groups were analyzed by ANOVA followed by the Student-Newman-Keuls test. Statistical significance was set at P < 0.05.

## Results

### Transplanted OPCs produce myelin sheath but not neurons and astrocytes

**Fig 1:**
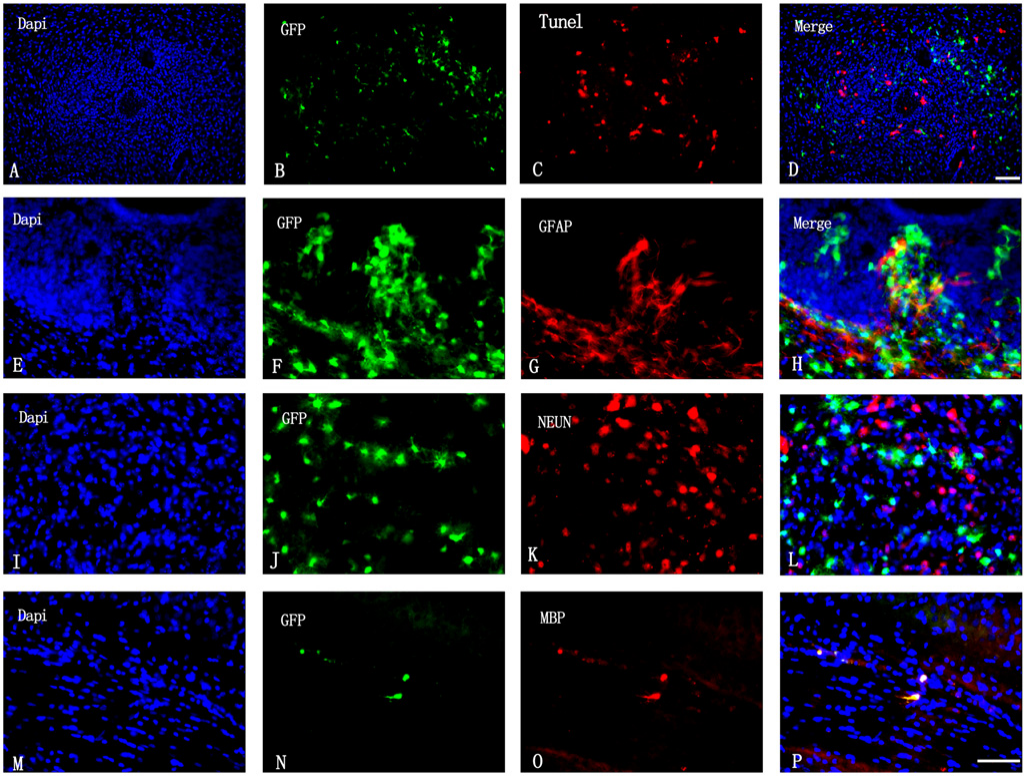
Survival and differentiation of transplanted OPCs. (A–D) Apoptosis of OPCs was analyzed labeling cells with DAPI (blue), GFP (green), and by the TUNEL reaction (red). Scale bar = 50 μm. (E–H) OPC differentiation into astrocytes was visualized by DAPI staining (blue) and GFP (green) and GFAP (red) immunolabeling. (I–L) OPC differentiation into neurons was visualized by DAPI staining (blue) and GFP (green) and NeuN (red) immunolabeling. (M–P) Myelin sheath formed by GFP^+^ OPCs was visualized by DAPI staining (blue), and GFP (green) and MBP (red) immunolabeling. Scale bar = 20 μm (E–P).

The TUNEL assay was used to assess the survival of transplanted cells 6 weeks after transplantation. To evaluate OPC differentiation, cells were double-immunolabeled for GFP/GFAP (astrocyte), GFP/NeuN (neuron), and GFP/MBP (myelin). There were few GFP^+^/TUNEL^+^ cells in the transplanted area (Fig. 1A-D); OPCs survived (Fig. 1A-D) and formed a myelin sheath (Fig. 1M-P), but did not differentiate into astrocytes (Fig. 1E-H) or neurons (Fig. 1J-L).

### Transplanted OPCs

**Fig 2:**
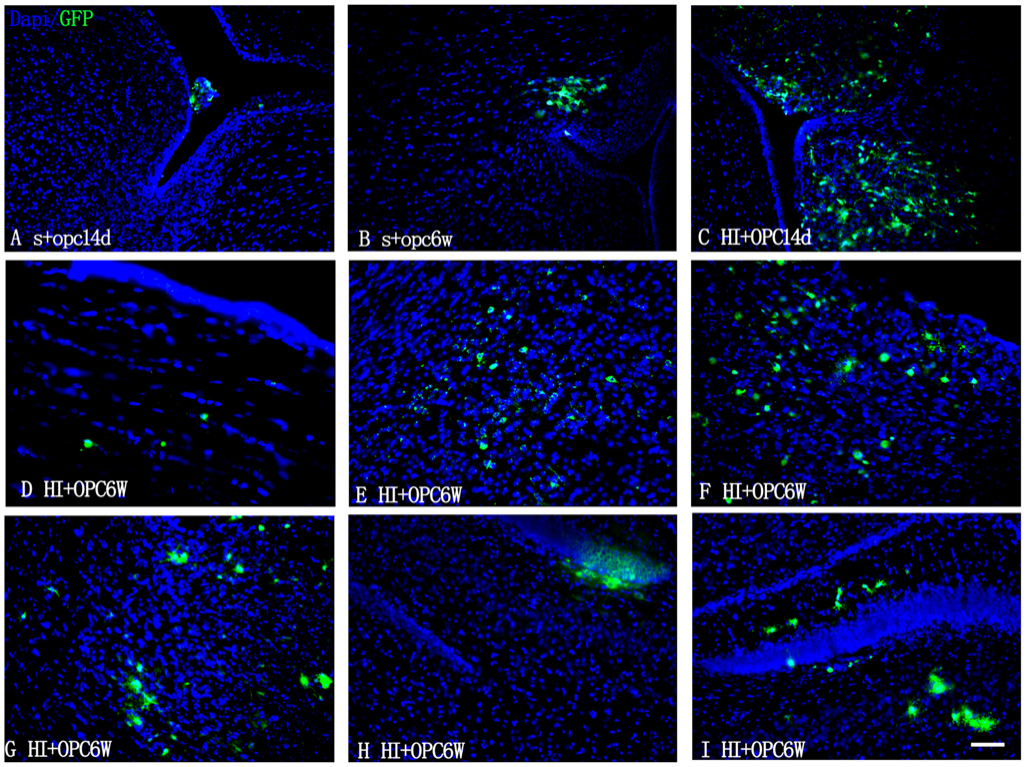
Migration of transplanted OPCs in the brain. GFP^+^ OPCs in the brains of rats subjected to sham operation or HI, then injected with OPCs. Animals were sacrificed 14 days and 6 weeks after transplantation, and coronal sections were immunostained for GFP and counterstained with DAPI. (A, C) GFP-expressing tranplanted OPCs in lateral ventricles and SVZ 14 days after transplantation. (B, D, F) GFP^+^ cells were observed in the regions surrounding the ventricles 6 weeks after transplantation. GFP^+^ OPCs were detected in (E, G) the cortex ispilateral to the side of injury and in the septofimbrial nucleus of the hippocampus and (H, I) in the SGZ of the hippocampus. Scale bar = 50 μm.

To determine if transplanted OPCs can penetrate the blood-brain barrier (BBB) and migrate into the parenchyma of the brain, the GFP^+^ cells were identified by immunolabeling 14 days and 6 weeks after transplantation. GFP^+^ cells were observed in the lateral ventricles 14 days after transplantation (Fig. 2A); in the HI+OPC group, many cells had migrated into the subventricular zone (SVZ) (Fig. 2C). At 6 weeks after transplantation, GFP^+^ cells were detected in the corpus callosum (Fig. 2D) and within the periventricular white matter, but were also observed migrating along the white matter tract of the cingulum around the periphery of the enlarged ventricles (Fig. 2B), confirming successful integration of transplanted cells into the host brain. GFP^+^ OPCs were also present in the injured ispilateral cortex (Fig. 2E), and the septofimbrial nucleus (Fig. 2F, G) and subgranular zone (SGZ) of the hippocampus (Fig. 2H, I).

### Stimulation of endogenous NSC proliferation by OPC transplantation in SVZ and dentate gyrus (DG) following HI

**Fig 3:**
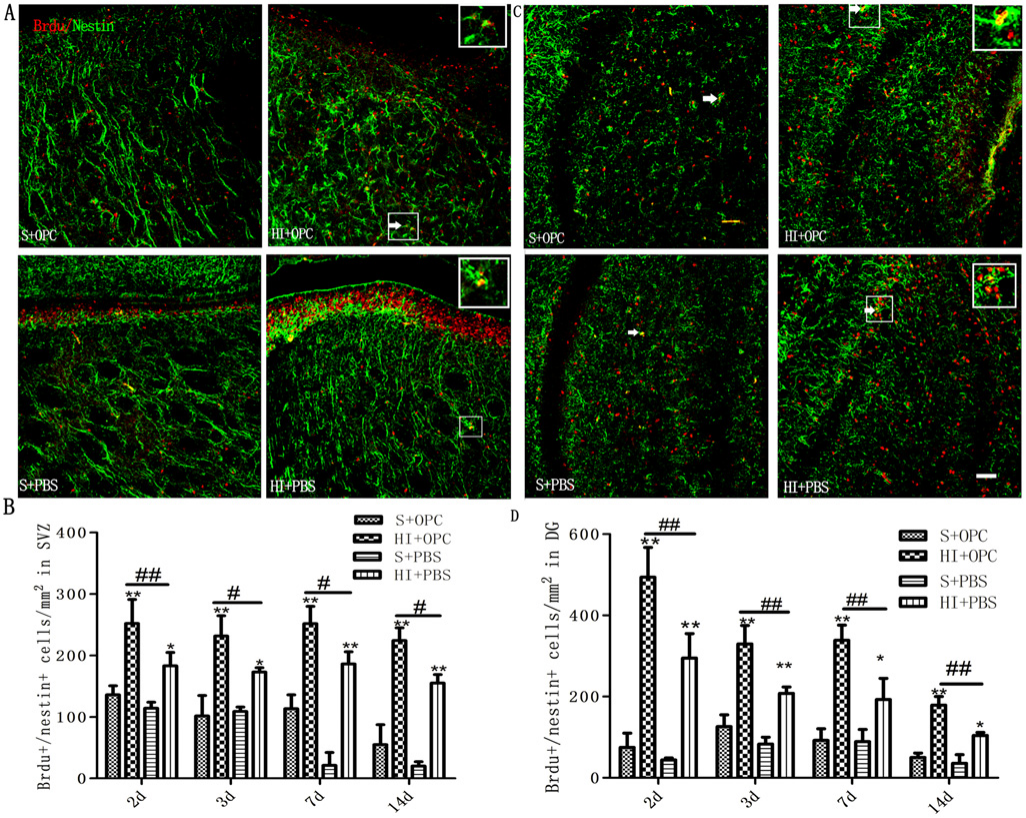
OPC transplantation promotes NSC proliferation in the SVZ and the DG of the hippocampus following HI. (A, C) Proliferating NSCs (white arrows) were visualized by double immunolabeling with antibodies against BrdU (red) and Nestin (green). (B, D) The number of BrdU^+^/Nestin^+^ proliferating NSC increased in HI animals (HI+PBS) compared to sham-operated controls; injection of OPCs (HI+OPC) potentiated this effect. Data are expressed as mean ± SD. *P < 0.05, **P < 0.01, HI group vs. sham controls; ^#^P < 0.05, ^##^P < 0.01 between HI+PBS and HI+OPC groups; n = 6–8 animals per group. Scale bar = 50μm.

To determine the effects of OPC transplantation on NSC proliferation following HI injury, animals were given daily injection of BrdU to label cycling cells until the time of sacrifice, at which time the brains were processed for combined immunohistochemical detection of BrdU and the NSC marker Nestin. Double-labeled cells in the two NSC niches, the SVZ (Fig. 3A, B) and DG of the hippocampus (Fig. 3C, D) were counted. HI injury stimulated proliferation of NSCs in both the SVZ and DG; the number of BrdU^+^-Nestin^+^ cells in the HI+OPC group was higher than in the HI+PBS control animals at 2, 3, 7, and 14 days after HI (Fig. 3B and 3D). We did not observe any effect of OPCs treatment on the number of BrdU^+^-Nestin^+^ cells in the contralateral hemisphere. These results indicate that OPCs transplantation can potentiate endogenous NSC proliferation following HI injury.

### Inhibition of HI-induced apoptosis by OPC transplantation in the DG

**Fig 4:**
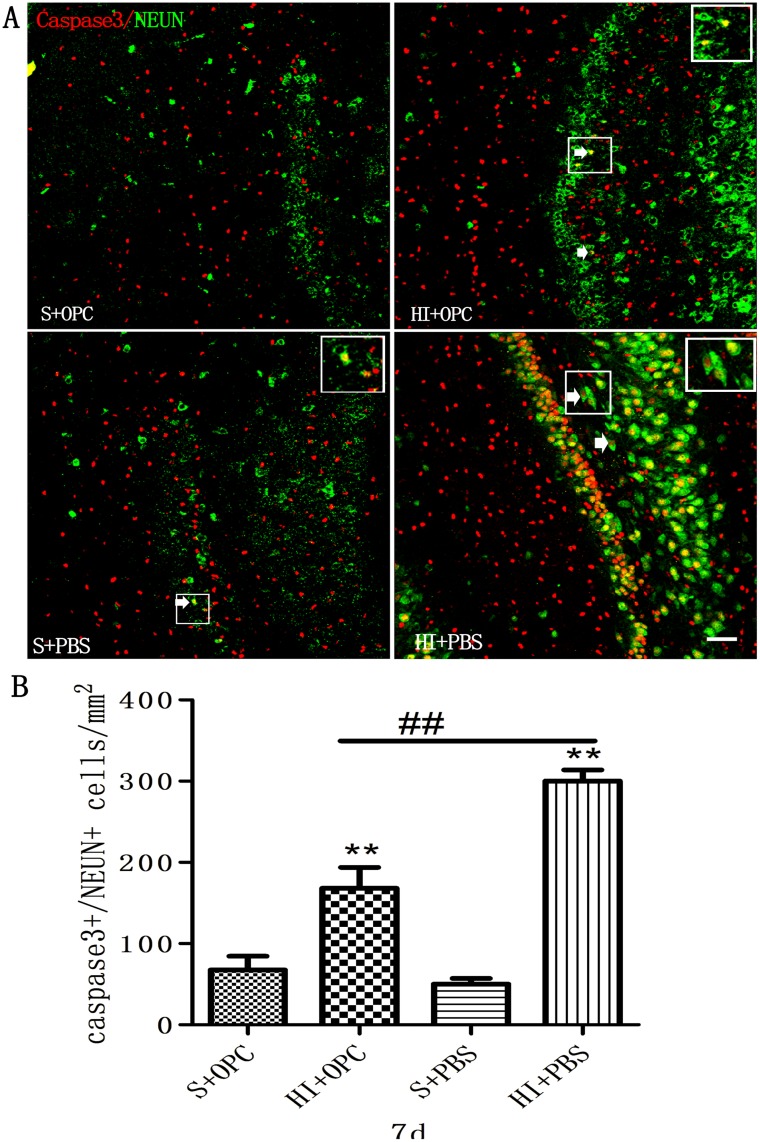
OPC transplantation inhibits apoptosis in the DG following HI 7 days after transplantation. (A) Apoptotic neurons (white arrows) were visualized by double immunolabeling with antibodies against caspase-3 (red) and NeuN (green). (B) The number of caspase-3^+^/NeuN^+^ apoptotic cells increased in HI animals (HI+PBS) compared to sham-operated controls; injection of OPCs (HI+OPC) mitigated this effect. Data are expressed as mean ± SD. *P < 0.05, **P < 0.01, HI group vs. sham controls; ^#^P < 0.05, ^##^P < 0.01 between HI+PBS and HI+OPC groups; n = 6–8 animals per group. Scale bar = 50 μm.

Given the effects on proliferation observed upon OPC transplantation, cell apoptosis was also assessed. To determine whether OPCs could decrease neuronal apoptosis induced by HI, cells were evaluated for caspase-3 and NeuN expression (Fig. 4A). HI injury resulted in a dramatic increase in apoptotic neurons in the DG after 7 days (Fig. 4B) compared to sham control animals. Transplantation of OPCs led to a nearly 50% decrease in the number of caspase-3^+^/NeuN^+^ cells (HI+OPC), although the number of apoptotic cells was still higher than in controls (S+OPC). These findings indicate that OPCs inhibit apoptosis of endogenous neurons following HI injury.

### OPC transplantation promotes BDNF and Bcl-2 expression in the cortex and SGZ after HI

**Fig 5:**
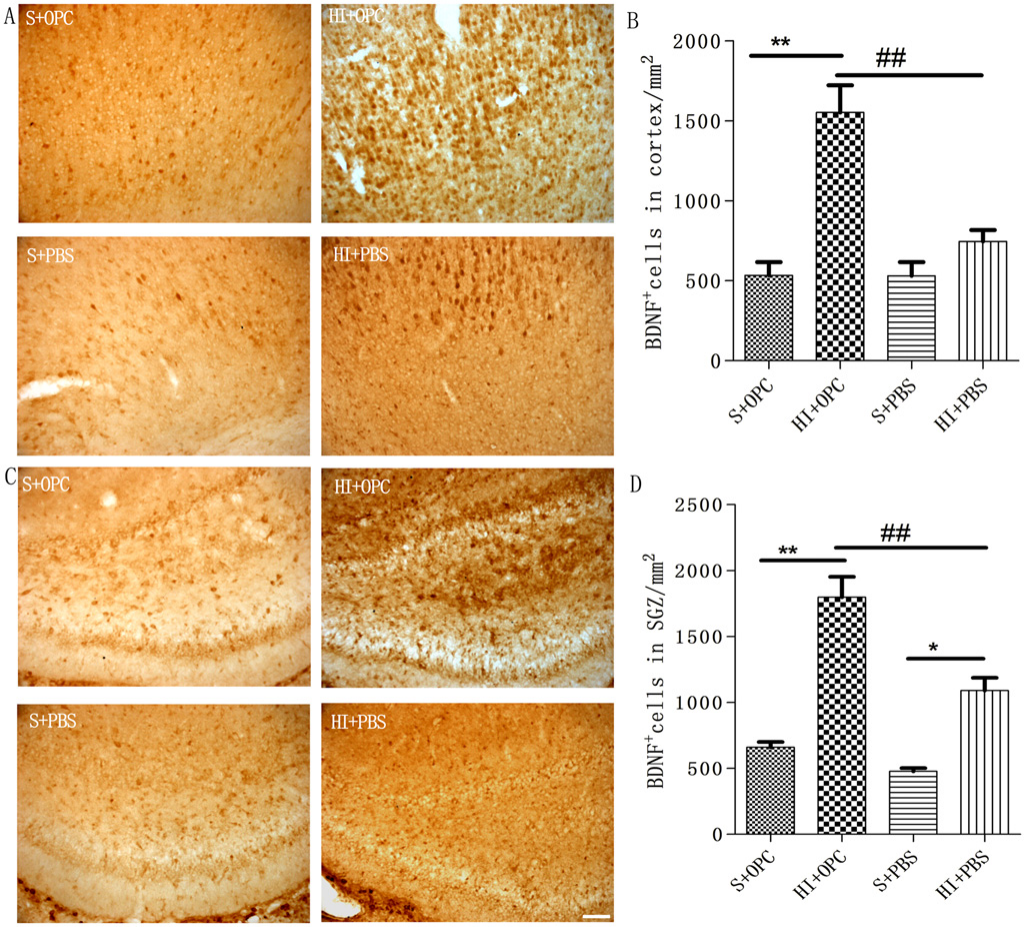
Stimulation of BDNF expression by transplanted OPCs. (A, C) BDNF^+^ cells were visualized by immunolabeling in the cortex and SGZ 7 days after transplantation. (B, D) The number of BDNF^+^ cells was increased in HI animals (HI+PBS) compared to sham-operated controls; injection of OPCs (HI+OPC) potentiated this effect. Data are expressed as mean ± SD. *P < 0.05, HI group vs. sham controls; ^#^P < 0.05, ^##^P < 0.01 between HI+PBS and HI+OPC groups; n = 6–8 animals per group. Scale bar = 50 μm.

**Fig 6:**
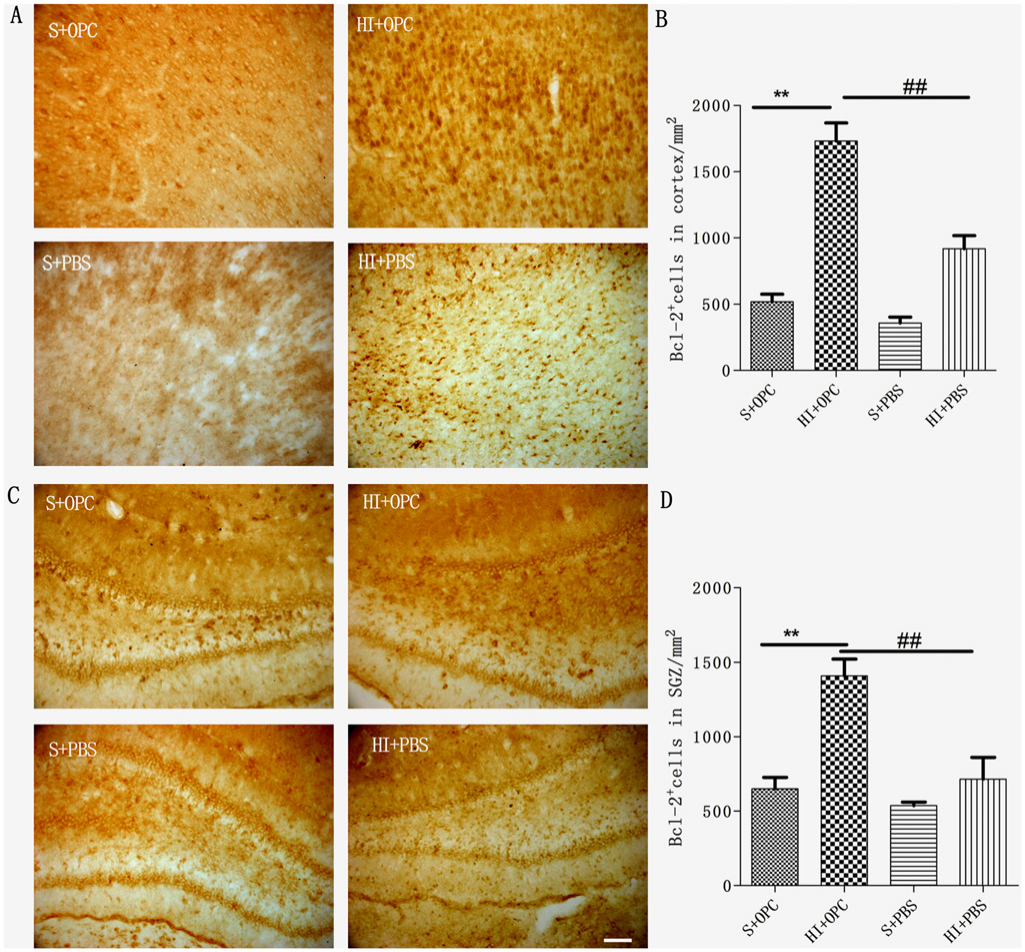
Stimulation of Bcl-2 expression by transplanted OPCs. (A, C) Bcl-2^+^ cells were visualized by immunolabeling in the cortex and SGZ 7 days after transplantation. (B, D) The number of Bcl-2^+^ cells increased, but not significantly, in HI animals (HI+PBS) compared to sham-operated controls; OPC injection (HI+OPC) potentiated this effect. Data are expressed as mean ± SD. ^#^P < 0.05, ^##^P < 0.01 between HI+PBS and HI+OPC groups; n = 6–8 animals per group. Scale bar = 50 μm.

To explore the mechanism underlying the neuroprotective effect of OPC transplantation, BDNF^+^ (Fig. 5A-D) and Bcl-2^+^ (Fig. 6A-D) cells in the cortex and SGZ were quantified 7 days after transplantation (Fig. 5A, C; Fig. 6A, C). BDNF expression increased after HI (Fig. 5B, D), but there was no significant difference in expression in the cortex of the sham group (Fig. 5B). Compared to the HI group, OPC transplantation stimulated BDNF secretion in the cortex and SGZ 7 days after transplantation (Fig. 5B-D). Similarly, Bcl-2 expression also increased after HI, with no difference observed in the cortex or SGZ of the sham group (Fig. 6B, D). Compared to the HI group, OPC transplantation stimulated the Bcl-2 expression in the cortex and SGZ 7 days after transplantation (Fig. 6B, D).

### Rescue of HI-induced spatial learning and memory deficits by OPC transplantation

**Fig 7:**
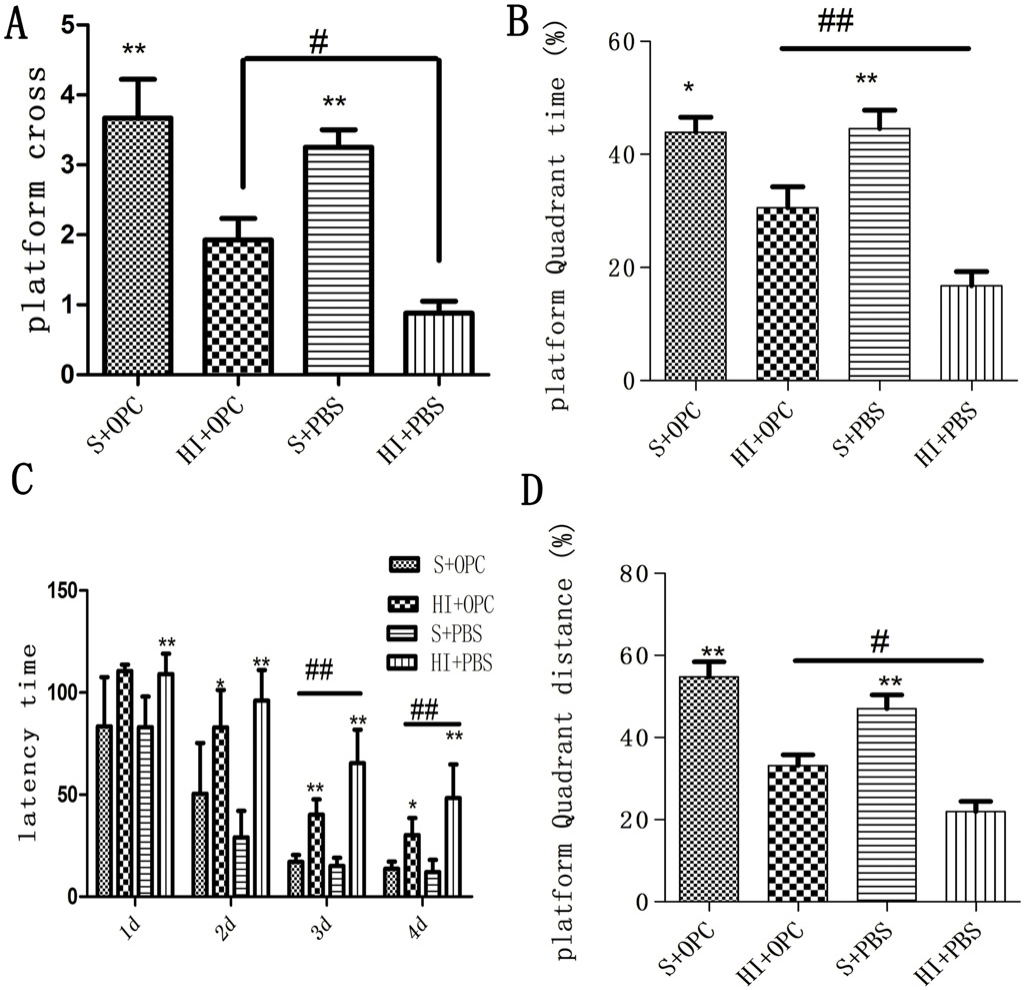
OPC transplantation improves spatial learning and memory deficits induced by HI. Animals were tested in the Morris water maze 36–40 days after transplantation. (A) Number of platform crossings; (B) percentage of time spent in the platform quadrant; (C) escape latency; (D) percentage of distance travelled in the platform quadrant. HI induced cognitive deficits in animals (HI+PBS), including longer escape latency, fewer platform crossings, and decreased time spent and distance traveled in the platform quadrant compared to sham animals; OPC transplantation partly reversed these effects. Data are expressed as mean ± SD. *P <0.05, **P < 0.01, HI group vs. sham controls. ^#^P < 0.05, ^##^P < 0.01 between HI+PBS and HI+OPC groups; n = 17 animals per group.

To assess the behavioral consequences of OPC transplantation, spatial learning and memory were tested in animals using the Morris water maze 36–40 days after transplantation. In the navigation trials, rats in the HI+PBS group showed an increased escape latency compared to controls, indicating that learning was impaired in these animals (Fig. 7C). However, HI+OPC animals had decreased escape latency compared to animals in the HI+PBS group. In the probe trails, HI+PBS animals had fewer platform crossings, a lower percentage of time spent in the target quadrant, and lower percentage of distance travelled in the target quadrant compared to sham control animals (Fig. 7A, B, D); these effects were partially rescued in the HI animals that received OPCs transplantation (HI+OPC) (Fig. 7A, B, D). Taken together, these results indicate that the cognitive deficits induced by HI injury can be reversed by the engraftment of OPCs into the injured area.

## Discussion

OPC transplantation was performed soon after birth, thereby providing timely intervention after HI-induced brain injury. Some preclinical studies have suggested its therapeutic effects in various CNS injury models, including leukodystrophies, multiple sclerosis, and transient symptoms of infarction in adult animals [22,26,27], but to date, there have been no studies applying OPC transplantation to brain injury in premature recipients. The present study investigated the therapeutic efficacy of OPC transplantation in a PVL model. Neonatal brain injury can be devided into two stages for treatment, the neuroprotective stage (within 24 hours of the insult), and the neurorestoration stage (beyond 24 hours after the insult)[28]. Researches have proven the time of transplantation not only at 3 hours but 3–10 days attenuate brain injury [29,30]. In newborns, a massive loss of neurons occurs in the first 24 h after HI injury [31]; for these reason, OPCs were injected into animals 2 h after HI so as to evaluate the beneficial effect of early intervention. This is because in clinical practice, OPCs would be introduced into the recipient newborn infants a few hours after birth, given the time that would be required to make a diagnosis.

Regional differences in oligodendrocyte differentiation in the adult brain have been observed upon OPC transplantation, with white matter promoting differentiation to a greater degree than gray matter [32]. To avoid obtaining ambiguous results, in this study the OPCs were injected into the lateral ventricle. OPCs rapidly respond to white matter injury and produce matrix metalloproteinase 9, which disrupts the BBB in the acute phase of brain injury [33]. The present results showed that OPCs injected into the ventricle can survive and migrate to the parenchyma of brain [7]. Cells can be delivered systemically or directly into the brain, such as intravascular [34], nasal administration [35], intracerebral injection [36] and intraperitoneal injection [30]. The intracranial route of administration is frequently used in rodent studies. The benefits is high dose of transplanted cells in the brain and short distance needing migrate, but for clinical application and risk of infection, less invasive routes are preferred. Intranasal delivery is a promising novel route to treat neonatal ischemic brain damage.

Compared to the HI+OPC groups, in the sham groups there was fewer GFP^+^ cells, and these migrated a shorter distance. Consistent with this finding, two studies have shown that OPCs have poor survival and fail to migrate following transplantation in the adult spinal cord [37,38]. Schwann cells also do not migrate extensively and have poor long-term survival when introduced into a normal CNS environment, which may be unfavorable to the foreign transplanted cells. OPCs could be detected in the brain 6 weeks after transplantation. Consistent with this finding, two studies showed OPCs could survive in the brain over longer periods of time up to 7–8 weeks after transplantation [7,22]. OPCs have been used in spinal cord injury and brain injury. They didn’t show the malignancies induced by transplantation [7,22,39]. In our study, we didn’t find tumor in the transplanted animals until 6 weeks. Malignancies need future study to highlight it.

OPCs differentiated into oligodendrocytes but not neurons or astrocytes, and formed a myelin sheath 6 weeks after transplantation, similar to results from previous investigations [7,22]; however, one study found that OPCs differentiated into astrocytes upon LPS-induce brain injury [7]. One possible reason for the disparity in their findings and ours is the stage of cells that were used: these authors employed oligodendrocyte precursor cells (early OPCs) is the first stage of oligodendrocyte lineage differentiation, which also have multipotent differentiated capacity than the OPCs (late OPCs) used here. Research indicated the environment of brain had influence on the differentiation of transplanted cells [40]. Differences in the transplanted cells’ environment attributable to the use of a different brain injury model and the time to detect the differentiation of OPCs may have also been another contributing factors.

In our study, OPC transplantation promoted the proliferation of endogenous NSCs and suppressed neuronal apoptosis. Umbilical cord blood cells also promote endogenous NSC proliferation, and decrease apoptosis in neurons after transplantion in HI-induced neonatal brain injury [12,13]. NSC transplantation had similar neuroprotective effects in a stroke model [41,42,43]. The results from OPCs presented here are consistent with the findings of another study using a lipopolysaccharide-induced inflammation model of brain injury. Transplanted OPCs also stimulated BDNF and Bcl-2 expression. BDNF is a neuroprotective factor that inhibits neuronal apoptosis along with Bcl-2; BDNF also improves learning and memory deficits in HI encephalopathy [44,45]. Cell transplantation strategies have traditionally been used to replace lost and damaged cells through targeted cellular differentiation; however, it is becoming increasingly apparent that exogenously transplanted cells, or endogenous cells stimulated *in situ*, are capable of a ‘bystander’ effect, providing neuroprotection through mechanisms independent of promoting differentiation [46]. In addition, OPCs secrete soluble factors that support neuronal survival [47,48,49], which could play a role in stimulating endogenous mechanisms of brain repair.

In this study, rats were tested in the Morris water maze to assess the behavioral consequences of OPCs transplantation. As expected, HI injury produced deficiencies in spatial learning and memory [50], which were partly mitigated by early OPC transplantation. This implies that OPCs not only replaced cells that were lost to injury, but stimulated BDNF and Bcl-2 expression to promote NSC proliferation and decrease neuronal apoptosis, possibly contributing to the rewiring of neuronal circuitry that resulted in improved cognitive function. However, additional work is needed to elucidate the detailed mechanism of how transplanted cells mediate functional recovery after brain injury.

In summary, in this PVL model, transplanted OPCs not only survived, migrated, and differentiated to form a myelin sheath, but provided neuroprotection by stimulating BDNF and Bcl-2 expression and the proliferation of endogenous NSCs while inhibiting neuronal apoptosis. These results can potentially be used to develop therapeutic strategies to minimize damage caused by HI-induced brain injury, although the mechanisms underlying the neuroprotective effects of OPCs require further investigation.

## References

[1] Zhu LH, Bai X, Zhang N, Wang SY, Li W, et al (2014) Improvement of human umbilical cord mesenchymal stem cell transplantation on glial cell and behavioral function in a neonatal model of periventricular white matter damage. Brain Res. 10.1016/j.brainres.2014.03.030 24680746

[2] Volpe JJ (2009) Brain injury in premature infants: a complex amalgam of destructive and developmental disturbances. Lancet Neurol 8: 110–124. 10.1016/S1474-4422(08)70294-1 19081519 PMC2707149

[3] Tuzun F, Gencpinar P, Ozbal S, Dilek M, Ergur BU, et al (2012) Neuroprotective effect of neotrofin in a neonatal rat model of periventricular leukomalacia. Neurosci Lett 520: 6–10. 10.1016/j.neulet.2012.04.076 22579826

[4] Blumenthal I (2004) Periventricular leucomalacia: a review. EUROPEAN JOURNAL OF PEDIATRICS 163: 435–442. 15179510 10.1007/s00431-004-1477-y

[5] Inder TE, Warfield SK, Wang H, Huppi PS, Volpe JJ (2005) Abnormal cerebral structure is present at term in premature infants. PEDIATRICS 115: 286–294. 15687434 10.1542/peds.2004-0326

[6] Pierson CR, Folkerth RD, Billiards SS, Trachtenberg FL, Drinkwater ME, et al (2007) Gray matter injury associated with periventricular leukomalacia in the premature infant. Acta Neuropathol 114: 619–631. 17912538 10.1007/s00401-007-0295-5 PMC2080348

[7] Webber DJ, van Blitterswijk M, Chandran S (2009) Neuroprotective effect of oligodendrocyte precursor cell transplantation in a long-term model of periventricular leukomalacia. Am J Pathol 175: 2332–2342. 10.2353/ajpath.2009.090051 19850891 PMC2789608

[8] Schultheiss TE, Kun LE, Ang KK, Stephens LC (1995) Radiation response of the central nervous system. Int J Radiat Oncol Biol Phys 31: 1093–1112. 7677836 10.1016/0360-3016(94)00655-5

[9] Franklin RJ, Ffrench-Constant C (2008) Remyelination in the CNS: from biology to therapy. Nat Rev Neurosci 9: 839–855. 10.1038/nrn2480 18931697

[10] Franklin RJ, Kotter MR (2008) The biology of CNS remyelination: the key to therapeutic advances. J Neurol 255 Suppl 1: 19–25. 10.1007/s00415-008-1004-6 18317673

[11] Zheng T, Weiss MD (2013) Neonatal transplant in hypoxic injury. Methods Mol Biol 1059: 147–156. 10.1007/978-1-62703-574-3_13 23934841

[12] Wang XL, Zhao YS, Hu MY, Sun YQ, Chen YX, et al (2013) Umbilical cord blood cells regulate endogenous neural stem cell proliferation via hedgehog signaling in hypoxic ischemic neonatal rats. Brain Res 1518: 26–35. 10.1016/j.brainres.2013.04.038 23632377

[13] Rosenkranz K, Kumbruch S, Tenbusch M, Marcus K, Marschner K, et al (2012) Transplantation of human umbilical cord blood cells mediated beneficial effects on apoptosis, angiogenesis and neuronal survival after hypoxic-ischemic brain injury in rats. Cell Tissue Res 348: 429–438. 10.1007/s00441-012-1401-0 22526623

[14] Obenaus A, Dilmac N, Tone B, Tian HR, Hartman R, et al (2011) Long-term magnetic resonance imaging of stem cells in neonatal ischemic injury. Ann Neurol 69: 282–291. 10.1002/ana.22168 21387373 PMC3069664

[15] Xia G, Hong X, Chen X, Lan F, Zhang G, et al (2010) Intracerebral transplantation of mesenchymal stem cells derived from human umbilical cord blood alleviates hypoxic ischemic brain injury in rat neonates. J Perinat Med 38: 215–221. 10.1515/JPM.2010.021 20121545

[16] Windrem MS, Nunes MC, Rashbaum WK, Schwartz TH, Goodman RA, et al (2004) Fetal and adult human oligodendrocyte progenitor cell isolates myelinate the congenitally dysmyelinated brain. Nat Med 10: 93–97. 14702638 10.1038/nm974

[17] Mothe AJ, Tator CH (2008) Transplanted neural stem/progenitor cells generate myelinating oligodendrocytes and Schwann cells in spinal cord demyelination and dysmyelination. Exp Neurol 213: 176–190. 10.1016/j.expneurol.2008.05.024 18586031

[18] Chen CP, Kiel ME, Sadowski D, McKinnon RD (2007) From stem cells to oligodendrocytes: prospects for brain therapy. Stem Cell Rev 3: 280–288. 18060584 10.1007/s12015-007-9006-9

[19] Erceg S, Ronaghi M, Oria M, Rosello MG, Arago MA, et al (2010) Transplanted oligodendrocytes and motoneuron progenitors generated from human embryonic stem cells promote locomotor recovery after spinal cord transection. Stem Cells 28: 1541–1549. 10.1002/stem.489 20665739 PMC2996083

[20] Du ZW, Li XJ, Nguyen GD, Zhang SC (2006) Induced expression of Olig2 is sufficient for oligodendrocyte specification but not for motoneuron specification and astrocyte repression. Mol Cell Neurosci 33: 371–380. 17035043 10.1016/j.mcn.2006.08.007

[21] Sheldon RA, Chuai J, Ferriero DM (1996) A rat model for hypoxic-ischemic brain damage in very premature infants. Biol Neonate 69: 327–341. 8790911 10.1159/000244327

[22] Sun Y, Xu CC, Li J, Guan XY, Gao L, et al (2013) Transplantation of oligodendrocyte precursor cells improves locomotion deficits in rats with spinal cord irradiation injury. PLoS One 8: e57534 10.1371/journal.pone.0057534 23460872 PMC3583877

[23] Fang CZ, Yang YJ, Wang QH, Yao Y, Zhang XY, et al (2013) Intraventricular injection of human dental pulp stem cells improves hypoxic-ischemic brain damage in neonatal rats. PLoS One 8: e66748 10.1371/journal.pone.0066748 23799131 PMC3682969

[24] Vorhees CV, Williams MT (2006) Morris water maze: procedures for assessing spatial and related forms of learning and memory. Nat Protoc 1: 848–858. 17406317 10.1038/nprot.2006.116 PMC2895266

[25] Xiong M, Zhang T, Zhang LM, Lu SD, Huang YL, et al (2008) Caspase inhibition attenuates accumulation of beta-amyloid by reducing beta-secretase production and activity in rat brains after stroke. Neurobiol Dis 32: 433–441. 10.1016/j.nbd.2008.08.007 18805488

[26] Tirotta E, Carbajal KS, Schaumburg CS, Whitman L, Lane TE (2010) Cell replacement therapies to promote remyelination in a viral model of demyelination. J Neuroimmunol 224: 101–107. 10.1016/j.jneuroim.2010.05.013 20627412 PMC2919340

[27] Kondo Y, Duncan ID (2009) Transplantation of oligodendrocyte progenitor cells in animal models of leukodystrophies. Methods Mol Biol 549: 175–185. 10.1007/978-1-60327-931-4_12 19378203

[28] Borlongan CV, Weiss MD (2011) Baby STEPS: a giant leap for cell therapy in neonatal brain injury. Pediatr Res 70: 3–9. 10.1038/pr.2011.228 21659957 PMC3117246

[29] Donega V, van Velthoven CT, Nijboer CH, van Bel F, Kas MJ, et al (2013) Intranasal mesenchymal stem cell treatment for neonatal brain damage: long-term cognitive and sensorimotor improvement. PLoS One 8: e51253 10.1371/journal.pone.0051253 23300948 PMC3536775

[30] Pimentel-Coelho PM, Magalhaes ES, Lopes LM, DeAzevedo LC, Santiago MF, et al (2010) Human cord blood transplantation in a neonatal rat model of hypoxic-ischemic brain damage: functional outcome related to neuroprotection in the striatum. Stem Cells Dev 19: 351–358. 10.1089/scd.2009.0049 19296724

[31] Northington FJ, Ferriero DM, Graham EM, Traystman RJ, Martin LJ (2001) Early Neurodegeneration after Hypoxia-Ischemia in Neonatal Rat Is Necrosis while Delayed Neuronal Death Is Apoptosis. Neurobiol Dis 8: 207–219. 11300718 10.1006/nbdi.2000.0371

[32] Vigano F, Mobius W, Gotz M, Dimou L (2013) Transplantation reveals regional differences in oligodendrocyte differentiation in the adult brain. Nat Neurosci 16: 1370–1372. 10.1038/nn.3503 23995069

[33] Seo JH, Miyamoto N, Hayakawa K, Pham LD, Maki T, et al (2013) Oligodendrocyte precursors induce early blood-brain barrier opening after white matter injury. J Clin Invest 123: 782–786. 10.1172/JCI65863 23281396 PMC3561802

[34] Tajiri N, Acosta SA, Shahaduzzaman M, Ishikawa H, Shinozuka K, et al (2014) Intravenous transplants of human adipose-derived stem cell protect the brain from traumatic brain injury-induced neurodegeneration and motor and cognitive impairments: cell graft biodistribution and soluble factors in young and aged rats. J Neurosci 34: 313–326. 10.1523/JNEUROSCI.2425-13.2014 24381292 PMC3866490

[35] Donega V, van Velthoven CT, Nijboer CH, van Bel F, Kas MJ, et al (2013) Intranasal mesenchymal stem cell treatment for neonatal brain damage: long-term cognitive and sensorimotor improvement. PLoS One 8: e51253 10.1371/journal.pone.0051253 23300948 PMC3536775

[36] Fang CZ, Yang YJ, Wang QH, Yao Y, Zhang XY, et al (2013) Intraventricular injection of human dental pulp stem cells improves hypoxic-ischemic brain damage in neonatal rats. PLoS One 8: e66748 10.1371/journal.pone.0066748 23799131 PMC3682969

[37] OLeary MT, Blakemore WF (1997) Oligodendrocyte precursors survive poorly and do not migrate following transplantation into the normal adult central nervous system. JOURNAL OF NEUROSCIENCE RESEARCH 48: 159–167. 9130144

[38] Franklin R, Blakemore WF (1996) Transplanted CG4 cells (an oligodendrocyte progenitor cell line) survive, migrate, and contribute to repair of areas of demyelination in X-irradiated and damaged spinal cord but not in normal spinal cord. EXPERIMENTAL NEUROLOGY 137: 263–276. 8635541 10.1006/exnr.1996.0025

[39] Bambakidis NC, Miller RH (2004) Transplantation of oligodendrocyte precursors and sonic hedgehog results in improved function and white matter sparing in the spinal cords of adult rats after contusion. The spine journal: official journal of the North American Spine Society 4. 10.1016/j.spinee.2003.07.004 14749190

[40] Park KI, Hack MA, Ourednik J, Yandava B, Flax JD, et al (2006) Acute injury directs the migration, proliferation, and differentiation of solid organ stem cells: evidence from the effect of hypoxia-ischemia in the CNS on clonal “reporter” neural stem cells. Exp Neurol 199: 156–178. 16737696 10.1016/j.expneurol.2006.04.002

[41] Zhang P, Li J, Liu Y, Chen X, Kang Q, et al (2009) Human neural stem cell transplantation attenuates apoptosis and improves neurological functions after cerebral ischemia in rats. Acta Anaesthesiol Scand 53: 1184–1191. 10.1111/j.1399-6576.2009.02024.x 19650809

[42] Chen B, Gao XQ, Yang CX, Tan SK, Sun ZL, et al (2009) Neuroprotective effect of grafting GDNF gene-modified neural stem cells on cerebral ischemia in rats. Brain Res 1284: 1–11. 10.1016/j.brainres.2009.05.100 19520066

[43] Lee HJ, Kim MK, Kim HJ, Kim SU (2009) Human neural stem cells genetically modified to overexpress Akt1 provide neuroprotection and functional improvement in mouse stroke model. PLoS One 4: e5586 10.1371/journal.pone.0005586 19440551 PMC2679145

[44] Almli CR, Levy TJ, Han BH, Shah AR, Gidday JM, et al (2000) BDNF protects against spatial memory deficits following neonatal hypoxia-ischemia. Exp Neurol 166: 99–114. 11031087 10.1006/exnr.2000.7492

[45] Han BH, Holtzman DM (2000) BDNF protects the neonatal brain from hypoxic-ischemic injury in vivo via the ERK pathway. J Neurosci 20: 5775–5781. 10908618 10.1523/JNEUROSCI.20-15-05775.2000 PMC6772561

[46] Martino G, Pluchino S (2006) The therapeutic potential of neural stem cells. Nat Rev Neurosci 7: 395–406. 16760919 10.1038/nrn1908

[47] Wilkins A, Majed H, Layfield R, Compston A, Chandran S (2003) Oligodendrocytes promote neuronal survival and axonal length by distinct intracellular mechanisms: a novel role for oligodendrocyte-derived glial cell line-derived neurotrophic factor. J Neurosci 23: 4967–4974. 12832519 10.1523/JNEUROSCI.23-12-04967.2003 PMC6741206

[48] Wilkins A, Chandran S, Compston A (2001) A role for oligodendrocyte-derived IGF-1 in trophic support of cortical neurons. Glia 36: 48–57. 11571783 10.1002/glia.1094

[49] Wilkins A, Compston A (2005) Trophic factors attenuate nitric oxide mediated neuronal and axonal injury in vitro: roles and interactions of mitogen-activated protein kinase signalling pathways. J Neurochem 92: 1487–1496. 15748166 10.1111/j.1471-4159.2004.02981.x

[50] Huang Z, Liu J, Cheung PY, Chen C (2009) Long-term cognitive impairment and myelination deficiency in a rat model of perinatal hypoxic-ischemic brain injury. Brain Res 1301: 100–109. 10.1016/j.brainres.2009.09.006 19747899

